# Huddling remodels gut microbiota to reduce energy requirements in a small mammal species during cold exposure

**DOI:** 10.1186/s40168-018-0473-9

**Published:** 2018-06-08

**Authors:** Xue-Ying Zhang, Gansukh Sukhchuluun, Ting-Bei Bo, Qing-Sheng Chi, Jun-Jie Yang, Bin Chen, Lei Zhang, De-Hua Wang

**Affiliations:** 1 State Key Laboratory of Integrated Management of Pest Insects and Rodents, Institute of Zoology, Chinese Academy of Sciences, Beijing, 100101 China; 2 College of Life Science, Shandong Normal University, Ji’nan, 250014 China; 3 University of Chinese Academy of Sciences, Beijing, 100049 China; 4 Microbiome Research Center, Shandong Institutes for Food and Drug Control, Ji’nan, 250101 China

**Keywords:** Body temperature, Cold adaptation, Energy intake, Gut microbiota, Huddling, Thermogenesis

## Abstract

**Background:**

Huddling is highly evolved as a cooperative behavioral strategy for social mammals to maximize their fitness in harsh environments. Huddling behavior can change psychological and physiological responses. The coevolution of mammals with their microbial communities confers fitness benefits to both partners. The gut microbiome is a key regulator of host immune and metabolic functions. We hypothesized that huddling behavior altered energetics and thermoregulation by shaping caecal microbiota in small herbivores. Brandt’s voles (*Lasiopodomys brandtii*) were maintained in a group (huddling) or as individuals (separated) and were exposed to warm (23 ± 1 °C) and cold (4 ± 1 °C) air temperatures (*T*_a_).

**Results:**

Voles exposed to cold *T*_a_ had higher energy intake, resting metabolic rate (RMR) and nonshivering thermogenesis (NST) than voles exposed to warm *T*_a_. Huddling voles had lower RMR and NST than separated voles in cold. In addition, huddling voles had a higher surface body temperature (*T*_surface_), but lower core body temperature (*T*_core_) than separated voles, suggesting a lower set-point of *T*_core_ in huddling voles. Both cold and huddling induced a marked variation in caecal bacterial composition, which was associated with the lower *T*_core_. Huddling voles had a higher α and β-diversity, abundance of *Lachnospiraceae* and *Veillonellaceae*, but lower abundance of *Cyanobacteria*, *Tenericutes*, TM7, *Comamonadaceae*, and *Sinobacteraceae* than separated voles. Huddling or cold resulted in higher concentrations of short-chain fatty acids (SCFAs), particularly acetic acid and butyric acid when compared to their counterparts. Transplantation of caecal microbiota from cold-separated voles but not from cold-huddling voles induced significant increases in energy intake and RMR compared to that from warm-separated voles.

**Conclusions:**

These findings demonstrate that the remodeling of gut microbiota, which is associated with a reduction in host *T*_core_, mediates cold- and huddling-induced energy intake and thermoregulation and therefore orchestrates host metabolic and thermal homeostasis. It highlights the coevolutionary mechanism of host huddling and gut microbiota in thermoregulation and energy saving for winter survival in endotherms.

**Electronic supplementary material:**

The online version of this article (10.1186/s40168-018-0473-9) contains supplementary material, which is available to authorized users.

## Background

Social animals and humans have evolved cooperative behaviors to maximize their fitness in harsh environments [1–3]. Among these behaviors, it has been hypothesized that huddling is one of the most acceptable benefits for species living in temperate and cold habitats to reduce thermoregulatory costs, especially in the cold season [4, 5]. Huddling mediates social thermoregulation by reducing the surface to volume ratio and, consequently, reduces heat loss and energy expenditure of the animal [6, 7]. Studies in some wild species such as the social degus (*Octodon degus*) and short-tailed field voles (*Microtus agrestis*) demonstrated that huddling reduced resting metabolic rate (RMR) and energy intake and that these metabolic advantages of huddling increased with lowered ambient temperature and increased group size [8–10].

The coevolution of mammals with their microbial communities confers fitness benefits to both partners [11–13]. Small herbivores depend mainly on caecal microbiota to digest cellulose and detoxify plant toxins [11, 14, 15]. Microbial diversity has been reported to be influenced by host physiology [16–18] and such factors as season [19–21], altitude [22], diet [23, 24], photoperiod [25], and air temperature [26, 27]. Conversely, gut microbiota, by way of their metabolites and bacterial polysaccharides, can influence host energy metabolism [26, 27], behavior [28, 29], inflammation and immunity [30–32], homeostasis of enteric and central nervous systems [33–35], and aging [36, 37]. These functions of gut microbiota have been confirmed mainly by microbiota transplantation [15, 26, 38]. Social rodent species increase the frequency of communal huddling in winter [39] and, thus, are more likely to be coprophagous. Therefore, we hypothesize that huddling may be a strong force in shaping gut microbiota and, consequently, mediate host energetics and thermoregulation.

Brandt’s voles (*Lasiopodomys brandtii*), a small non-hibernating herbivorous rodent species, are widely distributed in Inner Mongolian grasslands of China, Mongolia, and Southeast Baikal region of Russia. Its habitat is characterized by extremely cold dry winter and deep frozen soil. Brandt’s voles show seasonal variations in energy metabolism and thermoregulation to adapt to the seasonal environments [40, 41]. They have evolved group-living strategies such as making sounds to warn each other of predators, hoarding food collectively, and huddling to survive the cold winter. Group size in one colony increases from about 10 in summer to 20 individuals in winter [42]. However, the benefits of huddling in energy metabolism in different seasons and the underlying physiological mechanisms remain largely unknown. Here, we investigated the changes in energy intake, thermogenesis, body temperature, and gut microbiota induced by huddling in Brandt’s voles in warm and cold environments, and we transplanted microbiota to examine the hypothesis that microbiota in huddling voles can reduce energy expenditure. We demonstrated that huddling alters gut microbiota to reduce energy intake and thermoregulatory responses in a small mammal species during cold exposure.

## Methods

### Animal experiments

Adult Brandt’s voles used in this study were from a laboratory breeding colony at the Institute of Zoology, Chinese Academy of Sciences (CAS). After weaning (at 3 weeks of age), the voles were housed with the same sex siblings at a light regime of 16-h light:8-h dark (lights on from 4:00 to 20:00) and room temperature of 23 ± 1 °C. The voles were fed a standard rabbit pellet chow (containing 18% protein, 3% fat, 12% fiber, and 47% carbohydrate, Beijing KeAo Bioscience Co.) and were provided with water ad libitum. The animal procedures were approved by the Animal Care and Use Committee of Institute of Zoology, CAS.

A total of 124 female voles (aged 4 months) were placed in 31 cages (4 voles/cage) and were divided into 4 treatments: cold (at 4 ± 1 °C) huddling (CH, 7 cages), cold separated (CS, 7 cages), warm (at 23 ± 1 °C) huddling (WH, 8 cages), and warm separated (WS, 9 cages). Sibling voles were chosen preferentially and were acclimated to the cage (42 cm × 27 cm × 20 cm) for 3 weeks and then to the treatment conditions for another 3 weeks. The cages contained four equal compartments separated by stainless steel walls with small holes (6 mm in diameter). Openings (7 cm × 7 cm) in the walls allowed movement among the compartments. The openings were open for the huddling groups but closed for the separated groups, which had olfactory, visual, and vocal contacts. Individual voles were dyed on different positions of the body for identification.

### Body weight and energy intake

Voles were weighed at the beginning (day 0) and the end of (day 28) of the study using an electronic balance (Sartorius Model BL 1500, ± 0.1 g), and body weight change was calculated. Food was provided ad libitum and food intake and fecal output were measured for three consecutive days in the last week of acclimation [43]. Uneaten food and feces were collected after the 3 days, oven dried at 60 °C until constant mass, and separated manually. Water content of the food offered was calculated by drying samples at 60 °C until constant mass, and its gross energy was measured by bomb calorimetry (Parr1281 Instrument, USA). Gross energy intake was calculated by subtracting the gross energy of the uneaten food from the gross energy of the food offered.

### RMR and nonshivering thermogenesis (NST)

RMR, measured as oxygen consumption, was determined in huddling and separated voles at their acclimated temperatures of 4 and 23 °C at the end of the experiment. An open-circuit respirometry system (TSE labmaster, Germany) with an air flow rate of 3 L/minute was used. Voles were maintained in a 5.8 L transparent metabolic chamber (type II for rats) with double-layered meshes for the separation of voles during the measurements. The animals were allowed 1 h for acclimation to the conditions, and then recordings of O_2_ uptake were taken for 2 h. An average of a minimum of three consecutive stable readings was taken as the RMR.

NST was determined on individual animals at 25 °C on the day after RMR measurements. The volume of metabolic chamber was 2.7 L (type I for mice). The voles were injected subcutaneously with norepinephrine (NE) (Shanghai Harvest Pharmaceutical Co. LTD) at a dosage of 2.53M_b_^-0.4^ (mg/kg; M_b_, body mass) [44]. NST was estimated as the four highest consecutive stable readings of oxygen consumption during 1 h.

### Body temperature

In the last week of acclimation, surface body temperature (*T*_surface_) was read with an infrared camera (FLIR E60, UK) from a distance of 40 cm, and the data were analyzed by FLIR Tools software [8, 26]. *T*_surface_ was averaged from seven to eight images for each cage. The perimeter temperature (*T*_p_) of the voles was determined by fitting a polygon around the individual animal in the case of separated animals and around the entire group for huddling animals using the option “isotherm” of the software. The highest temperature in an image was selected as the maximum temperature (*T*_m_). Furthermore, the contact temperature (*T*_c_) between two animals in the huddling groups was determined by the “mobile” option.

Core body temperature (*T*_core_) was recorded during the acclimation period through transponders (G2 E-Mitter, to ± 0.1 °C, STARR life sciences) implanted in the abdomen of one vole in each cage (*n* = 4–5 voles/group). The voles were anesthetized by intraperitoneal injection of pentobarbital sodium (50 mg/kg). After the abdominal skin was sterilized with iodophor, an incision of up to 1 cm in length was made below the diaphragm. The wound was closed with absorbable PGA surgical suture (Jinhuan Model R413, 4/0) and sterilized with iodophor again. The animals were allowed 10 days to recover from surgery [45]. All receivers for collecting data were connected to a computer with the Vital View software.

### Short-chain fatty acids (SCFAs)

At the end of experiment, one vole from each cage (but not the one with E-Mitter) was sacrificed by CO_2_ asphyxiation. The digestive tract and caecal content were removed on a super-clean worktable, frozen immediately in liquid nitrogen, and stored at − 80 °C. Six SCFAs including acetic, propionic, butyric, isobutyric, valeric, and isovaleric acids were measured in caecal contents by high-performance gas chromatography (GC, Agilent 7890A; Agilent Technologies, Germany) with a GC autosampler and a FID system by the modified method [46, 47]. Caecal contents were extracted directly with water and did not require derivatization [46, 47]. Separations were performed in a 30 m × 0.25 mm × 0.25 μm DB-WAX column (Agilent Technologies) using 99.998% hydrogen as carrier gas at a flow rate of 1.0 mL/min. The system was operated at 250 °C. Injections were performed in the splitless mode at 230 °C, and 0.5 μL for each injection. The oven temperature was programmed from 60 °C (1 min) to 200 °C at 5 °C/min and then from 200 to 230 °C at 10 °C/min. The total running time of each sample was 32 min.

### Microbiota DNA extraction, evaluation, and amplification

DNA from caecal contents was extracted by 2 × CTAB (cetyltrimethyl ammonium bromide), phenol chloroform mixture (phenol:chloroform:isoamyl alcohol = 25:24:1). In the later steps of DNA isolation, we used the spin column from SanPrep Column DNA Gel Extraction Kit (Sangon Biotech, China) (based on a silica gel membrane) to purify and recover the DNA rapidly. DNA concentration was measured by fluorometry using the Qubit® dsDNA high-sensitivity assay kit and the Qubit® 2.0 fluorometer (Life Technologies, Carlsbad, CA, USA) as instructed by the manufacturer. DNA purity was also assessed by absorbance on a Nanodrop 2000 (Thermo Fisher Scientific, Carlsbad, CA, USA) by measuring the A260/A280 ratio. Only DNAs with an A260/A280 ratio of 1.8–2.0 were used for PCR amplification (*n* = 6 voles/group). Our target was the V3–V4 hyper-variable region of the bacterial 16S rRNA gene. PCR was started immediately after the DNA was extracted. The 16S rRNA V3–V4 amplicon was amplified using 2 × Taq PCR MasterMix (Tiangen, Beijing, China). Two universal bacterial 16S rRNA gene amplicon PCR primers (PAGE purified) were used: forward primer-341F (CCTACGGGNGGCWGCAG) and reverse primer-805R (GACTACHVGGGTATCTAATCC) [48]. To multiplex the samples during sequencing, barcodes were added to the 5′ termini of the forward primers (Additional file 1 Table S1). The PCR reaction was set up as follows: template DNA 2 μL, amplicon PCR forward primer (10 μM) 1 μL, amplicon PCR reverse primer (10 μM) 1 μL, and 2 × Taq PCR MasterMix 12.5 μL (total 25 μL). PCR was performed for each DNA sample in triplicate in the same thermal cycler (T100™ BIO-RAD) using the following program: 1 cycle of denaturing at 94 °C for 5 min, followed by 34 cycles of denaturing at 96 °C for 30 s, annealing at 52 °C for 30 s, elongation at 72 °C for 30 s, and a final extension at 72 °C for 5 min. The PCR products were checked using electrophoresis in 1% (*w*/*v*) agarose gels in TBE buffer (Tris, boric acid, EDTA) stained with ethidium bromide (EB) and visualized under UV light. PCR products were pooled and purified using Agencourt AMPure XP magnetic beads (Beckman) according to the manufacturer’s instructions. Then Sequencing was done on an Illumina HiSeq 2500.

### 16S rRNA gene amplicon sequencing analysis

The 16S sequence paired-end data set was joined and quality filtered using the FLASH method described by Magoč and Salzberg [49]. All sequences analysis was provided in the Quantitative Insights Into Microbial Ecology (QIIME, version 1.9.1) software suite [50], according to the Qiime tutorial (http://qiime.org/) with some modified methods. Chimeric sequences were removed using usearch61 [51] with de novo models. Sequences were clustered against the 2013 Greengenes (13_5 release) ribosomal database’s 97% reference data set. Sequences that did not match any entries in this reference were subsequently clustered into de novo OTUs at 97% similarity with UCLUST. Taxonomy was assigned to all OTUs using the RDP classifier [52] within QIIME and the Greengenes reference data set. Rarefaction and rank abundance curves were calculated from OTU tables using alpha diversity and rank abundance scripts within the QIIME pipeline. The hierarchical clustering based on population profiles of most common and abundant taxa was performed using UPGMA clustering (Unweighted Pair Group Method with Arithmetic Mean, also known as average linkage) on the distance matrix of OTU abundance. This resulted in a Newick formatted tree, which was obtained utilizing the QIIME package.

### Caecal microbiota transplantation (CMT)

To remove microbiota, the healthy 4-month-old male voles were housed in separate cages at a room temperature (23 ± 1 °C) and were offered water with fresh composite antibiotics (containing 100 μg/mL neomycin, 50 μg/mL streptomycin, 100 U/mL penicillin; Sigma, Germany) for 7 days as described previously [26]. For microbiota transplantation, the caecal contents were collected from three donors each of CH, CS, and WS male voles, diluted (200 mg) in 0.9% sodium chloride injection (physiological saline, 2 mL), and then, a 200 μL suspension was delivered by intragastric gavage to each bacteria-restricted recipient vole (*n* = 6 voles/group). For the control group, saline (200 μL) was delivered by intragastric gavage to each animal. The recipients were still housed in separate cages at room temperature after CMT. Body weight and energy intake were measured during antibiotic treatment and within 1 week after CMT, and RMR and NST were determined 1 week after CMT. After the experiment, the voles were sacrificed and caecal contents were collected for 16S rRNA sequencing (*n* = 2–4/group) and SCFAs measurement.

### Statistical analysis

The software SPSS 17.0 was used for statistical analyses. Prior to statistical analyses, all the data were examined for assumptions of normality and homogeneity of variance by Kolmogorov–Smirnov and Levene tests, respectively. The data with abnormal distribution were transformed by natural logarithms to normalize them. The data for body weight gain were transformed by arcsine and analyzed by two-way ANOVA (cold and huddling). Energy intake, RMR and NST were analyzed by two-way ANCOVA with body weight as a covariate. *T*_surface_, *T*_core_, the concentration of SCFAs, and microbial composition were analyzed by two-way ANOVA. Significant group differences were further evaluated using Bonferroni post hoc tests. All values were presented as mean ± SEM (standard error of mean), and *P* < 0.05 was considered to be statistically significant.

For microbiota data, to account for any bias caused by uneven sequencing depth, the least number of sequences present in any given sample from a sample category was selected randomly prior to calculating community-wide dissimilarity measures (α-diversity and β-diversity). We rarefied the OTU table to a sequencing depth of 12,000 per sample for both diversity analyses. All principal coordinate analyses (PCoA) were based on unweighted and weighted UniFrac distances using evenly sampled OTU abundances. Significance for PCoA (β-diversity) analyses was checked with multivariate permutation tests using the nonparametric method “ADONIS” (999 permutations) included in the package “vegan” of the QIIME-incorporated version of “R”. The boxplot representation of the α-diversity results was done with STAMP [53] and the calculation of *P* values was done with Welch’s *t* test. The linear discriminant analysis (LDA) Effect Size (LEfSe) method was used to assess differences in microbial communities using a LDA score threshold of 2 [54].

## Results

### Body weight gain, energy intake, RMR, and NST

**Fig. 1:**
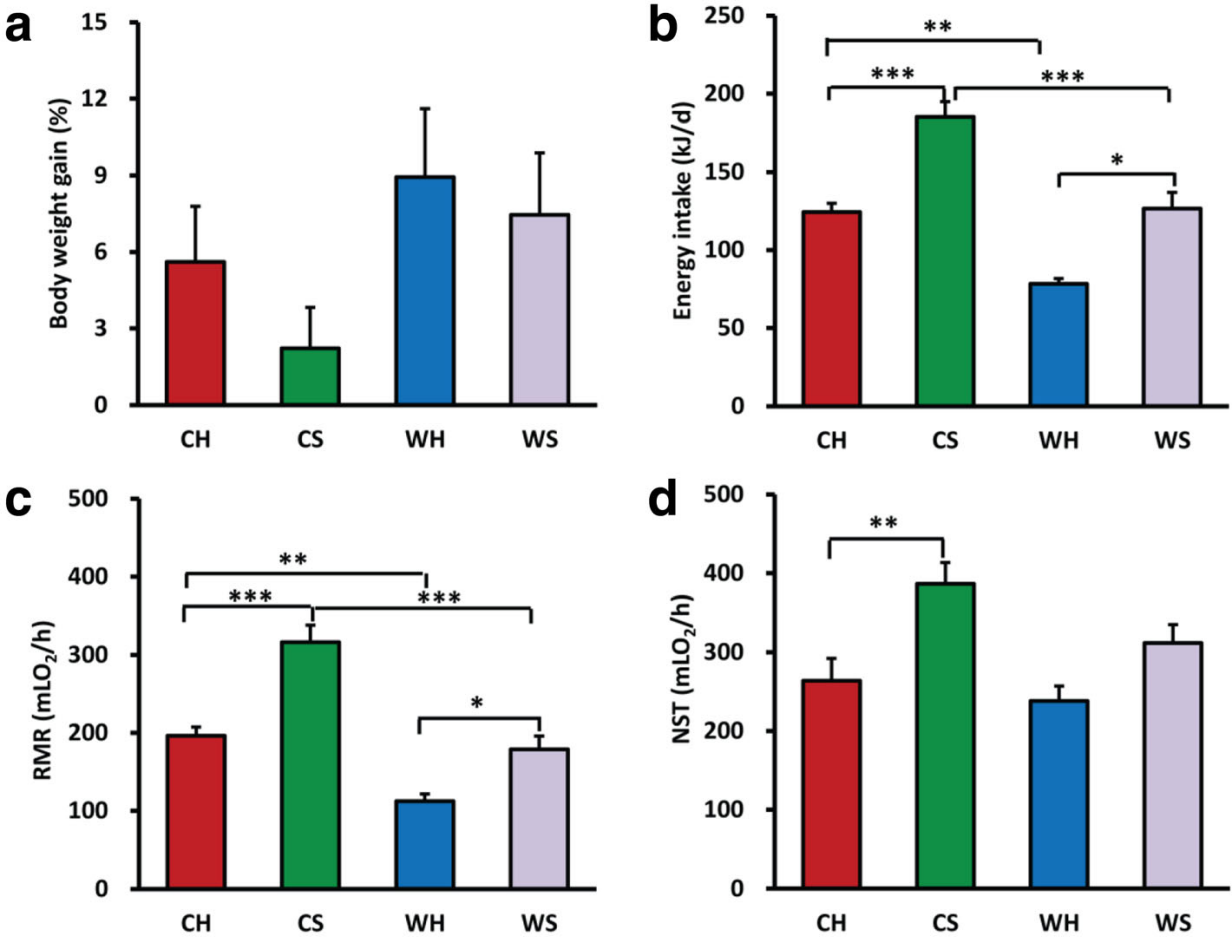
Effects of huddling and cold on metabolic phenotypes. **a**–**d** Body weight gain, energy intake (*n* = 6–8/group), resting metabolic rate (RMR), and nonshivering thermogenesis (NST) (*n* = 7–9/group) in huddling and separated Brandt’s voles at warm and cold air temperatures (*T*_a_). *P < 0.05, **P < 0.01, and *** *P* < 0.001

Body weight at the end (day 28) of acclimation was not affected by huddling or cold (Additional file 1Table S2). The voles in cold air temperature (*T*_a_) tended to gain less body weight than those in warm (*F*_1,64_ = 3.467, *P* = 0.067). Body weight gain was not affected by huddling (*F*_1,64_ = 1.120, *P* = 0.294) or the interaction between cold and huddling (*F*_1,64_ = 0.174, *P* = 0.678; Fig. 1a). Cold led to a significant increase in energy intake compared with warm condition (*F*_1,22_ = 46.259, *P* < 0.001; Fig. 1b). The huddling voles consumed less energy than separated voles in cold and warm conditions (*F*_1,22_ = 28.433, *P* < 0.001). Energy intake was not affected by the interaction between cold and huddling (*F*_1,22_ = 1.352, *P* > 0.05).

At the end of experiment, huddling voles had lower RMR than separated voles (*F*_1,26_ = 27.041, *P* < 0.001; Fig. 1c). RMR was affected by cold (*F*_1,26_ = 47.378, *P* < 0.001), but was not affected by the interaction between cold and huddling (*F*_1,26_ = 2.550, *P* = 0.122). RMR of huddling voles was 37% lower than in separated ones, both in cold (*P* < 0.001) and warm *T*_a_ (*P* = 0.05) (Fig. 1c). The huddling voles also had lower NST than separated voles (*F*_1,24_ = 8.458, *P* = 0.008; Fig. 1d). NST was marginally affected by cold (*F*_1,24_ = 3.815, *P* = 0.063), but was not affected by the interaction between cold and huddling (*F*_1,24_ = 0.928, *P* = 0.345). NST of CH voles was 31% lower than CS voles (*P* = 0.01), but was not different from WH voles (*P* > 0.05) (Fig. 1d).

### Body temperature

**Fig. 2:**
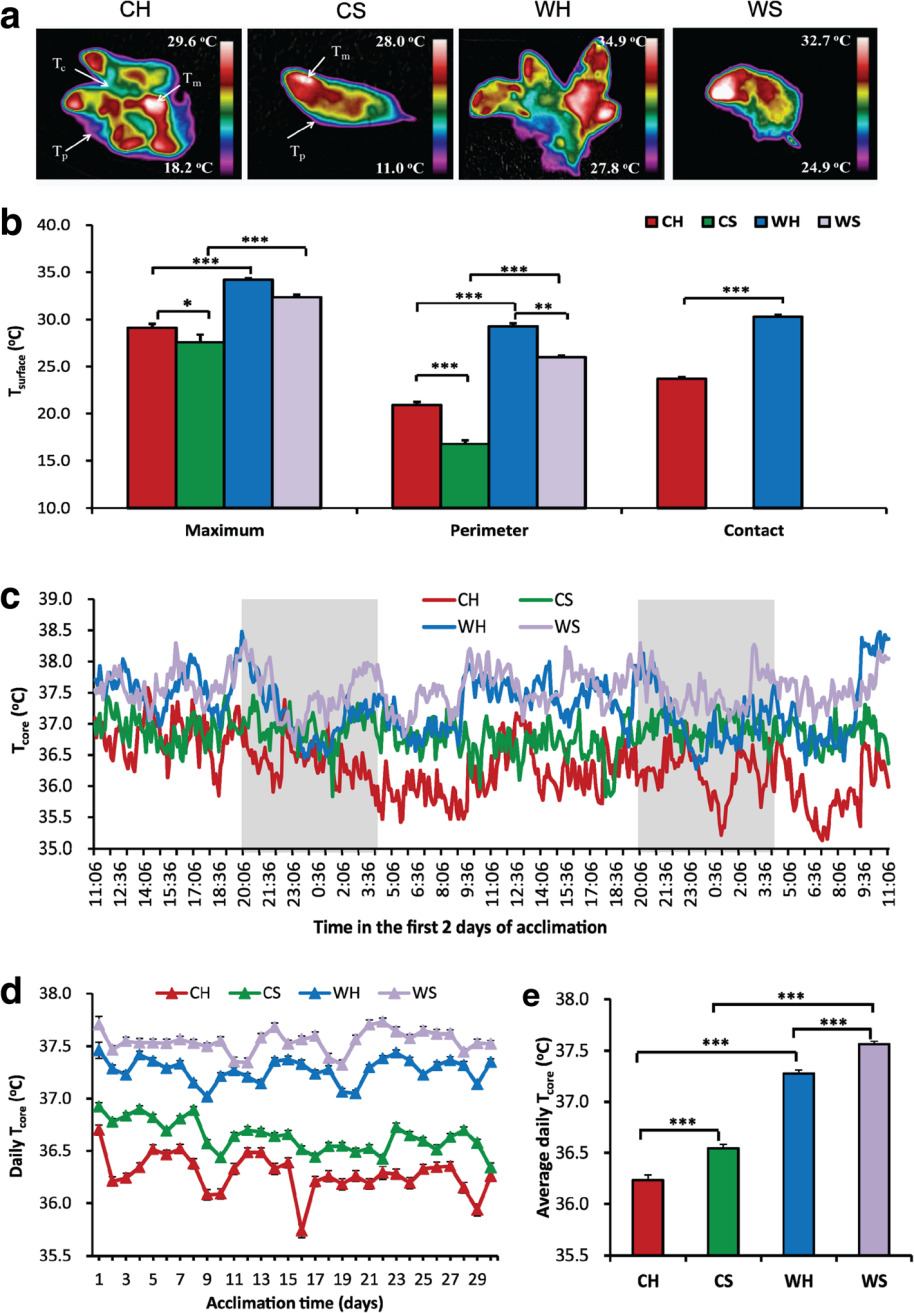
Effects of huddling and cold on body temperature. **a** Infrared images of representative CH (cold huddling), CS (cold separated), WH (warm huddling), and WS (warm separated) voles (*n* = 7–8/group) measured in the second week of acclimation. **b** *T*_surface_ (surface body temperature) by infrared temperature readings from the eye (maximum temperature, *T*_m_), around individual animals, or the entire groups (perimeter temperature, *T*_p_) and between two animals only in huddling groups (contact temperature, *T*_c_). **c** *T*_core_ (core body temperature) in the first 2 days of exposure (*n* = 4–5) in huddling and separated Brandt’s voles at warm and cold *T*_a_. The gray area indicated scotophase (20:00–04:00). **d**, **e** Average daily *T*_core_ (*n* = 4–5) in huddling and separated Brandt’s voles at warm and cold *T*_a_. *** *P* < 0.001

The *T*_surface_ including *T*_m_ and *T*_p_ was affected significantly by huddling (*P* < 0.001), cold (*P* < 0.001) and their interaction (*P* < 0.05; Fig. 2a, b). Huddling voles had higher *T*_m_ and *T*_p_ than separated voles both in warm (by 2 °C) and cold (by 3.1 °C for *T*_m_ and 4.4 °C for *T*_p_) conditions. Cold exposure resulted in lower *T*_m_ (by 4.6 °C in huddling and by 5.8 °C in separated group) and *T*_p_ (by 8 °C in huddling and by 10.4 °C in separated group) than in warm *T*_a_. The *T*_c_ was 7.5 °C lower in cold huddling than in warm huddling voles (*t* = 10.917, *df* = 13, *P* < 0.001).

In the first 2 days of exposure to cold, *T*_core_ dropped immediately both in huddling and separated voles. The separated voles in cold increased *T*_core_ to the same level to the voles in warm, while the huddling voles continued to decrease *T*_core_ (Fig. 2c). After 10 days of exposure, the voles kept relatively stable *T*_core_ in cold *T*_a_. Cold-exposed voles decreased their daily *T*_core_ by 1 °C compared with voles in warm *T*_a_ (*F*_1,52_ = 994.295, *P* < 0.001) (Fig. 2d, e). *T*_core_ was 0.3 °C lower in huddling voles than separated voles both in warm and cold conditions (*F*_1,52_ = 91.581, *P* < 0.001). The daily *T*_core_ was not affected by the interaction between cold and huddling (*F*_1,52_ = 0.500, *P* > 0.05).

### Huddling and cold alter the diversity and composition of gut microbiota

**Fig. 3:**
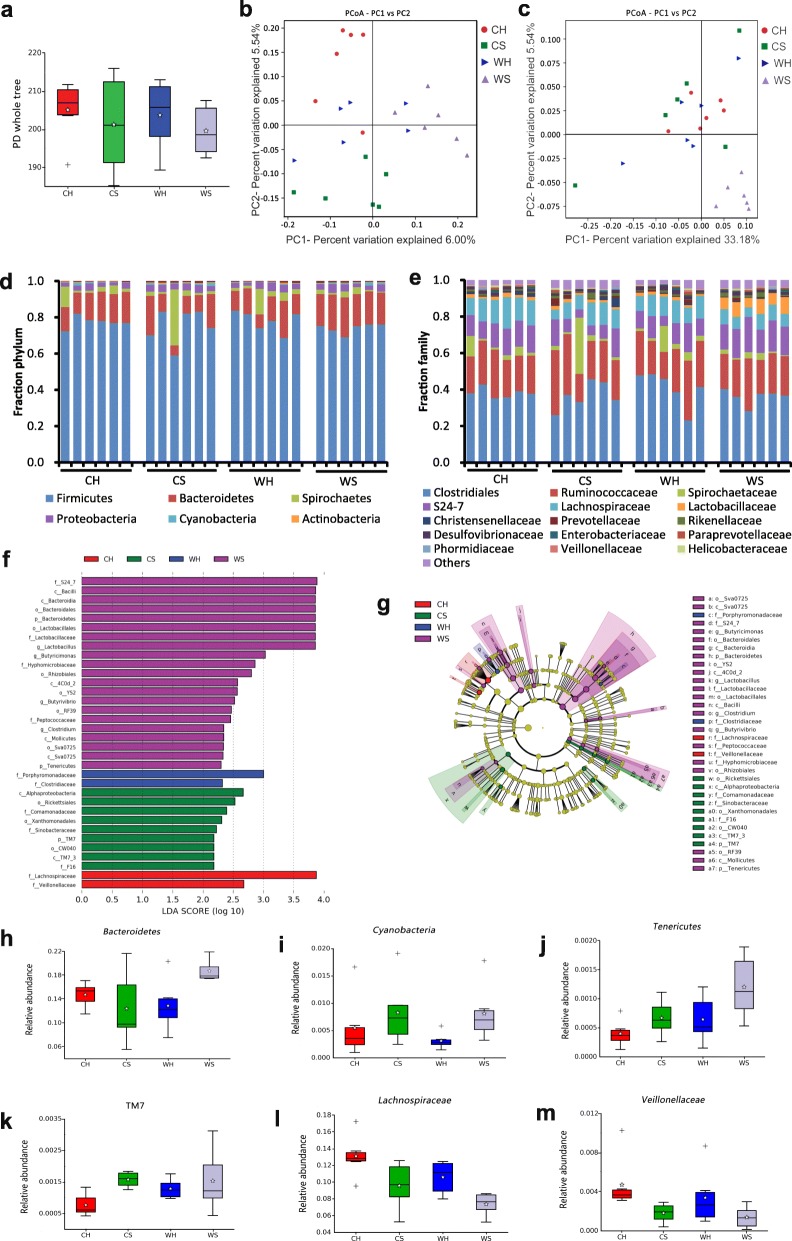
Both huddling and cold alter the diversity and composition of caecal microbiota. **a** Phylogenetic diversity (PD)—whole tree analysis for the samples from CH (cold huddling), CS (cold separated), WH (warm huddling), and WS (warm separated) voles after 3 weeks of acclimation. **b**, **c** Principal coordinates analysis (PCoA) plots based on unweighted and weighted UniFrac distance. Each symbol represents a single sample of caecal contents from the four groups. **d**, **e** Relative abundance at phylum and family levels in caecal microbiota community of the four groups. **f** Differential bacterial taxonomy selected by LEfSe analysis with LDA score > 2 in caecal microbiota community of the four groups. **g** Cladogram representing taxa enriched in caecal microbiota community of the four groups detected by the LEfSe tool. Differences were represented by the color of the most abundant class (red indicating CH group, green CS group, blue WH group, purple WS group, and yellow non-significant). The diameter of each circle is proportional to the taxon’s abundance. **h**–**m** Relative abundance of *Bacteroidetes*, *Cyanobacteria*, *Tenericutes*, TM7, *Lachnospiraceae*, and *Veillonellaceae* in caecal microbiota community of the four groups. In panels **a** and **h**–**m**, the white star indicates the mean of data, and the whisker indicates the most extreme data point within 1.5*(75th–25th percentile) of the median. Data points outside of the whiskers are shown as crosses

Huddling voles had a higher phylogenetic diversity (α-diversity) than separated voles (*P* = 7.85e−4), whereas the effect of cold temperature on phylogenetic diversity was not significant (*P* = 0.938) (Fig. 3a, Additional file 1: Table S1, Additional file 1: Table S3). The PCoA of unweighted (Fig. 3b) and weighted (Fig. 3c) UniFrac distances (β-diversity) between each sample among four groups showed that the microbial communities were significantly separated by cold at the first principal coordinate (PC1 axis) except for the WH group and also by huddling at the second principal coordinate (PC2 axis) in the cold (Fig. 3b, Additional file 1: Table S4). There were significant differences in UniFrac distances within groups (*F*_7,196_ = 11.261, *P* < 0.001; Additional file 1: Figure S2). Intergroup UniFrac distances were markedly higher than intragroup distances (*P* < 0.001), indicating distinctive microbial community structures in each group. The intragroup distances were significantly affected by huddling (*F*_1,56_ = 4.903, *P* = 0.031) and the interaction of huddling and cold (*F*_1,56_ = 11.461, *P* = 0.001), but not affected by cold (*F*_1,56_ = 0.976, *P* = 0.328).

Analysis at the phylum level showed that the dominant phyla (mean relative abundance > 1%) in voles included *Firmicutes* (76.9%) and *Bacteroidetes* (13.6%). Rare phyla included *Spirochaetes* (4.9%), *Proteobacteria* (3.2%), *Cyanobacteria* (0.6%), *Actinobacteria* (0.4%), and TM7 (0.1%) (Fig. 3d). Analysis at the family level showed that the dominant six families were *Clostridiales* (38.7%), *Ruminococcaceae* (23.1%), S24–7 (11.1%), *Lachnospiraceae* (10.9%), *Spirochaetaceae* (4.9%), and *Lactobacillaceae* (1.9%) (Fig. 3e).

To assess differences in microbial communities affected by huddling and cold, we applied LEfSe method with LDA score >  2 (Fig. 3f, g). The results identified 2 discriminative features in the microbiota of CH voles, 9 in CS, 2 in WH, and 21 in WS voles. Huddling induced major shifts of the microbiota composition, with significant decreases in proportions of *Cyanobacteria*, *Tenericutes* and TM7 at the phylum level, and of RF39, *Rickettsiales*, *Comamonadaceae*, and *Sinobacteraceae* at the order or family level, but increases in *Lachnospiraceae* and *Veillonellaceae* when compared with the separated voles (Fig. 3f, h–m, Additional file 1: Table S5). The proportions of *Tenericutes*, RF39, *Lactobacillaceae*, *Peptococcaceae*, and *Clostridiaceae* were lower, but of *Lachnospiraceae* were higher in cold voles than in warm voles. The proportions of *Bacteroidetes* were affected by the interaction of cold and huddling and were higher in CH and WS groups than in CS and WH groups.

### Huddling and cold alter microbial metabolites

**Fig. 4:**
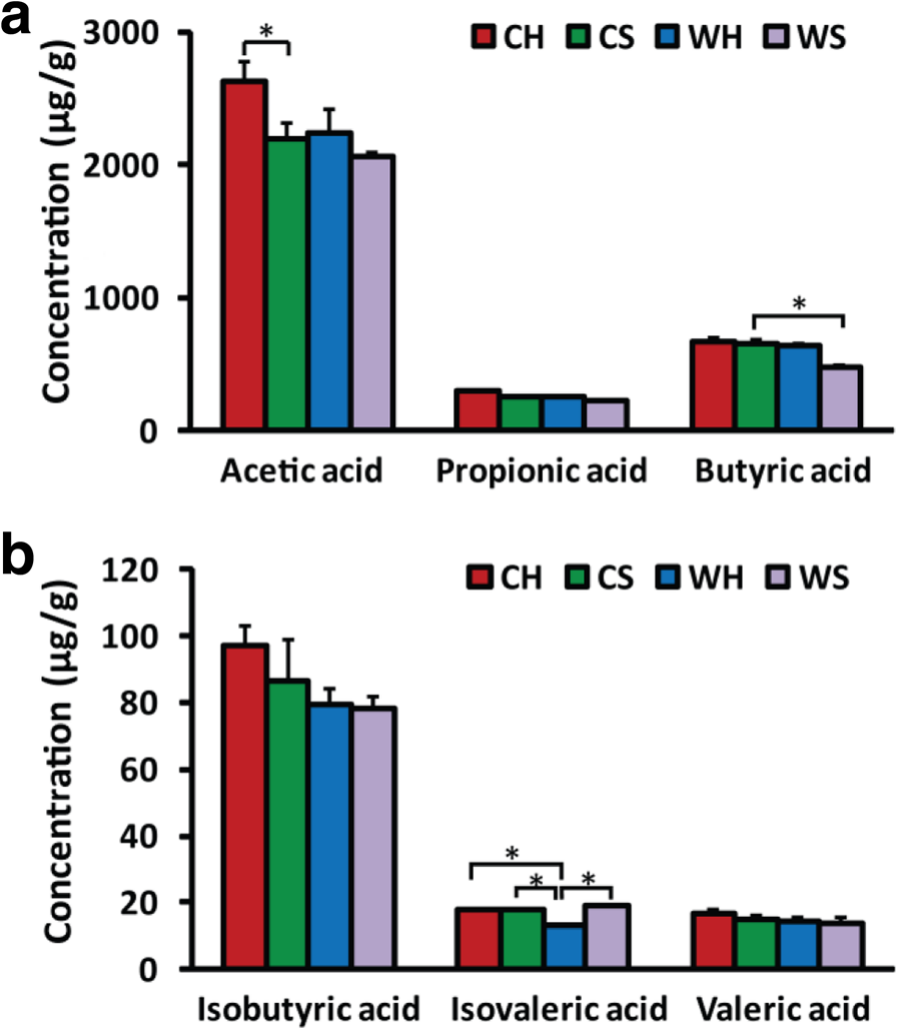
Effects of huddling and cold on the concentrations of short-chain fatty acids (SCFAs). The concentrations of acetic acid (**a**), propionic acid (**b**), butyric acid (**c**), isobutyric acid (**d**), valeric acid (**e**), and isovaleric acid (**f**) in huddling and separated Brandt’s voles at warm and cold *T*_a_ (*n* = 7–9/group). *P < 0.05

The huddling voles increased acetic acid concentration in cold (*F*_1,26_ = 6.256, *P* = 0.019), but decreased isovaleric acid concentration in warm (*F*_1,26_ = 6.311, *P* = 0.019) when compared with separated voles (Fig. 4). Cold exposure induced increases in the concentrations of acetic acid in huddling voles (*F*_1,26_ = 4.649, *P* = 0.041) and butyric acid in separated voles (*F*_1,26_ = 4.588, *P* = 0.042) when compared with warm conditions. The concentrations of propionic acid, isobutyric acid, and valeric acid were not affected by cold or huddling (*P* > 0.05). Total SCFAs were higher in cold than those in warm (*F*_1,26_ = 5.293, *P* = 0.030) and in huddling than those in separated voles (*F*_1,26_ = 5.710, *P* = 0.024). The isovaleric acid concentration was affected by the interaction between cold and huddling (*F*_1,26_ = 5.094, *P* = 0.033).

### CMT alters energy metabolism, gut microbiota, and microbial metabolites

**Fig. 5:**
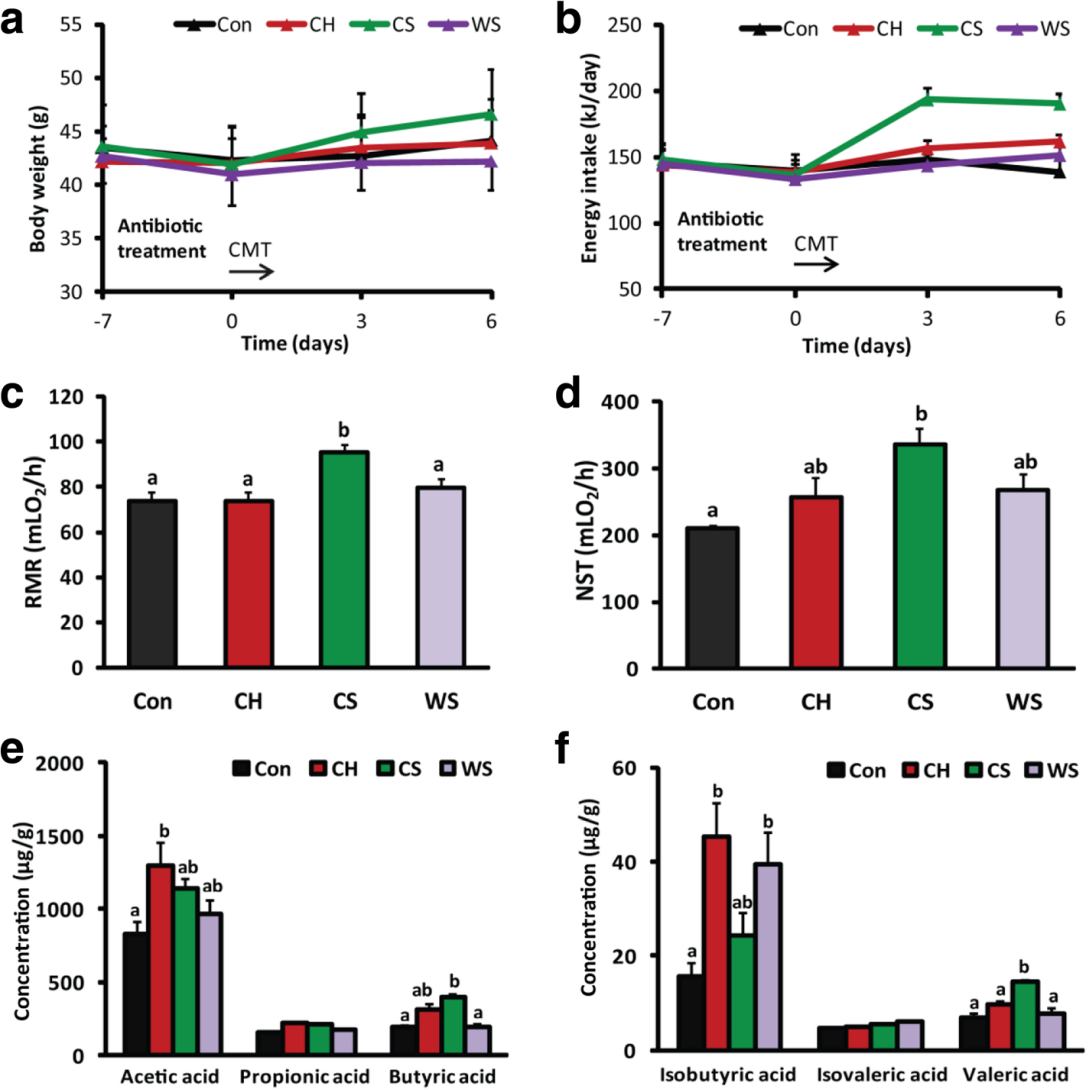
Caecal microbiota transplantation (CMT) affects energy metabolism and microbial metabolites. **a** There was no difference in body weight before or after CMT among groups (*n* = 6/group). **b** CS microbiota increased energy intake compared with CH and WS microbiota and Con (saline) after 3 days of CMT. There was no difference in energy intake among Con, CH, and WS groups. **c** CS rather than CH microbiota increased RMR compared with saline (Con) and WS microbiota. **d** CS rather than CH microbiota increased NST compared with Con voles. **e**, **f** CMT resulted in significant changes in the concentrations of acetic acid, butyric acid, isobutyric acid, and valeric acid among groups. Bars not sharing common letters indicate a significant difference (*P* < 0.05)

There was no difference in body weight among all groups during antibiotic treatment (*F*_3,20_ = 0.038, *P* = 0.990) or after CMT (*F*_3,20_ = 0.135, *P* > 0.05; Fig. 5a). Energy intake among groups did not differ during antibiotic treatment (*F*_3,19_ = 0.253, *P* = 0.871), but CMT from CS voles led to an increase in energy intake at day 3 (*F*_3,19_ = 10.253, *P* < 0.001) and day 6 (*F*_3,19_ = 8.914, *P* = 0.001; Fig. 5b) compared to the other groups. The voles transplanted with CS microbiota had higher RMR than the other three groups (*F*_3,19_ = 6.688, *P* = 0.003; Fig. 5c) and also had higher NST than the control group (*F*_3,19_ = 5.001, *P* = 0.010; Fig. 5d).

CMT resulted in significant changes in the concentrations of acetic acid (*F*_3,20_ = 3.802, *P* = 0.026), butyric acid (*F*_3,20_ = 4.265, *P* = 0.018), isobutyric acid (*F*_3,20_ = 5.909, *P* = 0.005), and valeric acid (*F*_3,20_ = 7.910, *P* = 0.001) among groups (Fig. 5e, f). Voles transplanted with CS microbiota had higher concentrations of butyric acid and valeric acid than the other groups (*P* < 0.05; Fig. 5e, f).

The data of 16S rRNA sequencing showed that antibiotic treatment and microbiota transplantation markedly altered microbial quantity as assessed by Shannon, chao1, observed OTUs, and PD whole tree (α-diversity, Additional file 1: Figure S3) and altered microbial community composition measured by unweighted UniFrac distances (β-diversity, Additional file 1: Figure S4 a–c). Group differences in microbial communities affected by antibiotic treatment and microbiota transplantation were found by LEfSe method with LDA score > 2 (Additional file 1: Figure S4 d). We also observed that the recipients (R) had the similar abundance of biomarkers as the donors (D) in the respective groups (Additional file 1: Figure S4 e–j).

## Discussion

Huddling is a social thermoregulatory behavior in mitigating cold stress in endotherms. In the present study, we investigated the underlying coevolutionary and physiological mechanisms of huddling in regulating energy intake and thermogenesis from the new aspect of gut microbiota. We observed that huddling voles had higher *T*_surface_ but lower *T*_core_ than voles that could not huddle (separated). Huddling also resulted in decreases in energy intake, RMR, and NST in both warm and cold *T*_a_. Both cold and huddling resulted in marked alterations in caecal bacterial composition at the phylum and family levels and also significant changes in microbial metabolites. Further, CMT indicated that huddling- or cold-induced variations in caecal microbiota regulate energy intake and metabolic rate of the host. These results suggest that the alteration in core temperature in the gut by huddling or cold may affect bacterial growth and activity and, hence, induce the observed changes in energy intake and thermoregulation.

### Huddling reduced metabolic costs

To maintain normal body temperature under cold *T*_a_, mammals use many physiological mechanisms, including increased heat production, heat conservation, and energy intake [55]. In small mammals, RMR and NST increased during cold exposure in Brandt’s voles [41, 56], prairie voles (*Microtus ochrogaster*) [57], and root voles (*Microtus oeconomus*) [58]. In the present study, although cold exposure induced an increase in RMR, huddling voles had a lower RMR than separated voles both at 4 and 23 °C. There is a lower RMR in huddling than in separated animals in many other wild mammals, such as social degus [8], short-tailed field voles [9], bank voles (*Clethrionomys glareolus*) [59], townsend’s voles (*Microtus townsendii*) [60], and African four-striped grass mice (*Rhabdomys pumilio*) [61]. Besides the lower RMR, the huddling voles did not increase NST in the cold indicating an effective social behavior for heat conservation in small mammals.

Due to the lower energy expenditure, the huddling voles consumed less energy than separated voles in both cold and warm *T*_a_. This was consistent with previous studies in mice (*Mus musculus*) [62], golden mice (*Ochrotomys nuttalli*) [63], furred Siberian hamsters (*Phodopus sungorus*) [64], and social degus at low *T*_a_ [8]. In contrast to warm T_a_, however, the voles in cold would gain less body weight and/or body fat (data not shown) due to high metabolic costs, which was also found in the previous studies in voles [41, 43]. The thermoneutral zone of Brandt’s vole ranges between 27.5 and 32.5 °C [65] and, therefore, the warm *T*_a_ of 23 °C used in this experiment was below the lower critical temperature. Therefore, huddling in warm group also had an energetic benefit.

### Huddling increased *T*_surface_ but decreased *T*_core_

Maintenance of high and constant *T*_core_ over a wide range of *T*_a_ is a high evolutionary feature of endotherms [66]. Huddling animals reduce their thermal conductance in cold through the reduced surface to volume ratio. In addition, the contact increases skin thickness in the area of contact and thus heat transfers between voles with less loss to the environment [9, 67]. The decrease in thermal conductance in huddling animals may reduce temperature loss to the environment and thus result in higher *T*_surface_ compared with separated animals. Further, the increased *T*_surface_ as warm resources may attract voles to huddle, especially in the cold.

We found that huddling voles had a lower *T*_core_ than separated voles, and CH voles had the lowest *T*_core_ among the four groups. A similar result of lower *T*_core_ under huddling has been recorded in birds where the goslings of greater snow geese (*Chen caerulescens atlantica*) in cold arctic environments had a *T*_core_ by 0.3 °C when huddling [68], and emperor penguin decreased *T*_core_ by 0.9 °C and increased local *T*_surface_ during huddling in pair period in Antarctic winters [69]. We assumed that, during huddling, the voles were resting and reduced their vigilance and control system of internal heat balance, which also allowed a gradual decrease in *T*_core_ to the possible minimum set-point of body temperature. Moreover, the decrease in *T*_core_ in response to a low *T*_a_ supports the importance of sensors of the thermal environment in determining the set-point to which *T*_core_ is regulated [70]. Other explanations for decreased *T*_core_ in huddling mostly came from the reduced metabolic rate in the present study and from previous studies [68, 69]. These data indicate that huddling contributes to the reduced set-point of *T*_core_ and temperature gradient between *T*_core_ and *T*_surface_ to reduce individual internal heat loss and conserve energy.

### Huddling shaped the diversity of caecal microbiota

Gut microbial diversity is vulnerable to the environments of wild mammals. For example, seasonal variations in gut microbial diversity were reported in wild wood mice (*Apodemus sylvaticus*) [19], ground squirrels (*Ictidomys tridecemlineatus*) [20], and wild black howler monkeys (*Alouatta pigra*) [21]. Gut microbiota also changed its diversity with different altitudes and diet diversity in plateau pikas (*Ochotona curzoniae*) [22], yaks (*Bos grunniens*), and Tibetan sheep (*Ovis aries*) [71]. We observed that huddling significantly increased the diversity of caecal microbiota compared with separated condition. Both cold and huddling could induce marked changes in caecal bacterial composition at the phylum and family levels, while huddling decreased the abundance of *Cyanobacteria*, *Tenericutes*, TM7, *Comamonadaceae*, and *Sinobacteraceae*. Most of these are pathogenic bacteria and have been found to be associated with host inflammatory mucosal diseases [72, 73]. Moreover, huddling increased the abundance of *Lachnospiraceae* and *Veillonellaceae*. The family *Lachnospiraceae* has been linked to protection from colon cancer mainly due to the production of butyric acid [74, 75]. The increased abundance of *Veillonellaceae* was previously found in the intestinal microbiota of breast-fed infants [76] and in human type 2 diabetes following prebiotic fiber intake [77], implying a beneficial effect due to the production of acetate and propionate. Therefore, these data indicate that huddling may shape the hosts to develop a healthier gut microbial community.

The shifts in caecal microbiota were associated with a reduction in *T*_core_ during cold exposure and in huddling in Brandt’s voles. Hibernating mammals exhibit an annual temperature rhythm [78, 79] and alter the diversity and composition of gut microbiota over the circannual hibernation cycle [20, 80]. Most mammals such as mice, desert hamsters (*Phodopus roborovskii*), and even humans show daily body temperature rhythm [81, 82] and the diurnal variation of gut microbiota has been reported in mice [83, 84]. A recent study showed that a 2–3 °C increase in ambient temperature can cause a 34% loss in gut bacterial diversity in a vertebrate ectotherm, the common lizard (*Zootoca vivipara*) [85]. Although there is still no direct evidence about the relationship between the host body temperature and gut microbiota in mammals, it is possible that a reduced core temperature in the gut may change bacterial growth and activity and, hence, the observed changes in gut microbial community.

### Gut microbiota regulates energy intake and thermogenic capacity of the host

Microbial metabolites, by interacting with enteric nervous system, provide the causal links between environment-induced alterations in gut microbiota and the physiological and behavioral responses of the host [32, 86]. We observed that the alteration in caecal microbiota was followed by changes in their metabolites. Cold exposure induced increases in concentrations of acetic acid in huddling voles and butyric acid in separated voles. A number of data indicated that the principal products of caecal fermentation of dietary fiber, such as acetic, butyric, and propionic acids, by acting on the free fatty acid receptors (FFAR2 and FFAR3, previously named G-protein-coupled receptors GPR43 and GPR41) contributed to regulating the release of anorexigenic hormones peptide YY (PYY) and glucagon-like peptide (GLP)-1 from the gut [87, 88] and leptin secretion from adipocytes [89], and controlled appetite, energy intake, and thermogenesis [90–92]. Thus, the increased SCFAs, particularly acetic acid and butyric acid in cold and/or huddling animals, help maintain metabolic and thermal homeostasis.

In the present study, the changes in energy intake, RMR and NST were accompanied by variations in caecal microbiota during cold exposure and during huddling. In addition, the voles transplanted with CH microbiota had a lower energy intake and RMR than voles transplanted with CS microbiota, confirming that gut microbiota regulated energy intake and metabolic rate of the host. Further support was provided by the findings that cold microbiota transplanted to germ-free mice led to an increase in white fat browning and energy expenditure [26, 27]. The concentrations of SCFAs also showed that CMT altered microbial metabolites, as CS microbiota increased the concentrations of acetic acid and butyric acid. These data provide further support for microbial functioning as a potential player in the shifted host metabolic physiology.

## Conclusion

**Fig. 6:**
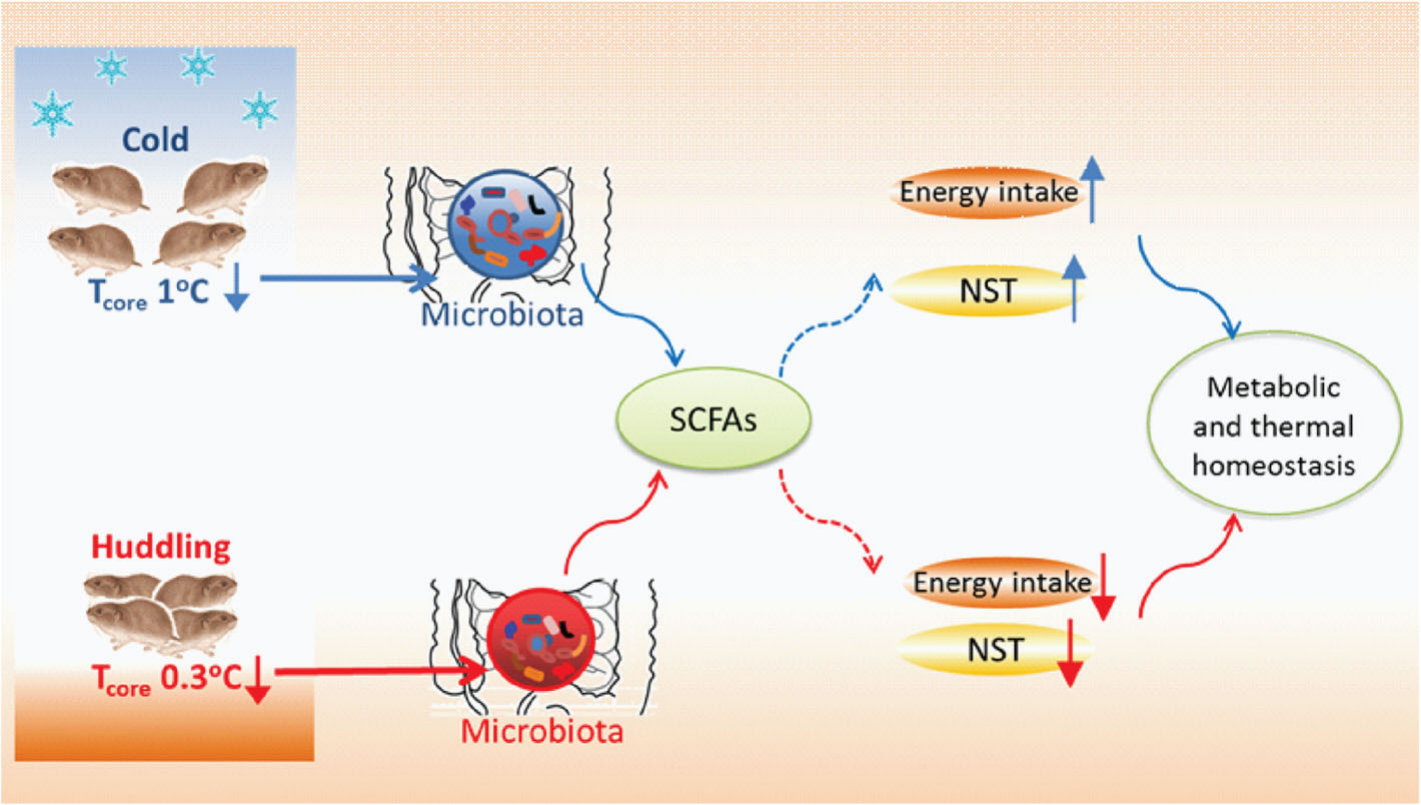
Schematic model that cold- or huddling-induced remodeling of gut microbiota orchestrates host metabolic and thermal homeostasis. SCFAs, short-chain fatty acids; NST, nonshivering thermogenesis

For the first time, the current study presents new insight into the coevolutionary mechanism of host huddling and gut microbiota in thermoregulation and energy saving for winter survival in endotherms. Huddling voles reduced energy intake and conserved energy by reducing metabolic rate and set-point of *T*_core_. A healthier gut microbial community was detected in huddling than in separated voles which produced more SCFAs, particularly in cold *T*_a_. Furthermore, CMT induced the alteration in energy intake, metabolic rate, microbial communities, and metabolites. These data demonstrate that the environment-associated reduction in host *T*_core_ may change the gut microbial community, and the remodeling of gut microbiota and their metabolites mediate cold- and huddling-induced energy intake and thermoregulation and therefore orchestrates host metabolic and thermal homeostasis (Fig. 6).

## Additional file

Additional file 1: Supplementary figures and tables. (DOC 1820 kb)
