# Correction to: Huddling remodels gut microbiota to reduce energy requirements in a small mammal species during cold exposure

**DOI:** 10.1186/s40168-018-0509-1

**Published:** 2018-07-09

**Authors:** Xue-Ying Zhang, Gansukh Sukhchuluun, Ting-Bei Bo, Qing-Sheng Chi, Jun-Jie Yang, Bin Chen, Lei Zhang, De-Hua Wang

**Affiliations:** 1 State Key Laboratory of Integrated Management of Pest Insects and Rodents, Institute of Zoology, Chinese Academy of Sciences, Beijing, 100101 China; 2 University of Chinese Academy of Sciences, Beijing, 100049 China; 3 College of Life Science, Shandong Normal University, Ji’nan, 250014 China; 4 Microbiome Research Center, Shandong Institutes for Food and Drug Control, Ji’nan, 250101 China

## Correction

Following publication of the original article [1], the authors reported an error in the caption of Fig. 4.

Originally, the incorrect figure caption was published as:

**Fig. 4** Effects of huddling and cold on the concentrations of short-chain fatty acids (SCFAs). The concentrations of acetic acid (**a**), propionic acid (**b**), butyric acid (**c**), isobutyric acid (**d**), valeric acid (**e**), and isovaleric acid (**f**) in huddling and separated Brandt’s voles at warm and cold *T*_a_ (*n* = 7–9/group). *P < 0.05

The correct caption is:

**Fig. 4** Effects of huddling and cold on the concentrations of short-chain fatty acids (SCFAs). The concentrations of acetic acid, propionic acid, butyric acid, isobutyric acid, valeric acid, and isovaleric acid in huddling and separated Brandt’s voles at warm and cold *T*_a_ (*n* = 7–9/group). *P < 0.05
