# Supplementary material for: Huddling remodels gut microbiota to reduce energy requirements in a small mammal species during cold exposure
Source: Microbiome. 2018 Jun 8;6:103. doi: 10.1186/s40168-018-0473-9 (PMC5994089; doi:10.1186/s40168-018-0473-9)
Supplement: Supplementary file 1 — Supplementary figures and tables. (DOC 1820 kb) [file 40168_2018_473_MOESM1_ESM.doc]

Table S1 Barcode sequence and primers

| Sample  ID | Barcode Sequence | Forward primer | Reverse primer |
| --- | --- | --- | --- |
| 2 | TAAGGCGACTCTCTAT | CCTACGGGNGGCWGCAG | GACTACHVGGGTATCTAATCC |
| 3 | TAAGGCGATATCCTCT | CCTACGGGNGGCWGCAG | GACTACHVGGGTATCTAATCC |
| 4 | TAAGGCGAGTAAGGAG | CCTACGGGNGGCWGCAG | GACTACHVGGGTATCTAATCC |
| 5 | TAAGGCGAACTGCATA | CCTACGGGNGGCWGCAG | GACTACHVGGGTATCTAATCC |
| 6 | TAAGGCGACTAAGCCT | CCTACGGGNGGCWGCAG | GACTACHVGGGTATCTAATCC |
| 7 | TAAGGCGAACACACTG | CCTACGGGNGGCWGCAG | GACTACHVGGGTATCTAATCC |
| 9 | TAAGGCGAACAGACAG | CCTACGGGNGGCWGCAG | GACTACHVGGGTATCTAATCC |
| 10 | TAAGGCGAACGAGATG | CCTACGGGNGGCWGCAG | GACTACHVGGGTATCTAATCC |
| 11 | TAAGGCGAACGTACGT | CCTACGGGNGGCWGCAG | GACTACHVGGGTATCTAATCC |
| 12 | TAAGGCGAACTCACAG | CCTACGGGNGGCWGCAG | GACTACHVGGGTATCTAATCC |
| 13 | CGTACTAGTAGATCGC | CCTACGGGNGGCWGCAG | GACTACHVGGGTATCTAATCC |
| 16 | CGTACTAGGTAAGGAG | CCTACGGGNGGCWGCAG | GACTACHVGGGTATCTAATCC |
| 17 | CGTACTAGACTGCATA | CCTACGGGNGGCWGCAG | GACTACHVGGGTATCTAATCC |
| 19 | CGTACTAGACACACTG | CCTACGGGNGGCWGCAG | GACTACHVGGGTATCTAATCC |
| 20 | CGTACTAGACACGTCA | CCTACGGGNGGCWGCAG | GACTACHVGGGTATCTAATCC |
| 21 | CGTACTAGACAGACAG | CCTACGGGNGGCWGCAG | GACTACHVGGGTATCTAATCC |
| 22 | CGTACTAGACGAGATG | CCTACGGGNGGCWGCAG | GACTACHVGGGTATCTAATCC |
| 24 | CGTACTAGACTCACAG | CCTACGGGNGGCWGCAG | GACTACHVGGGTATCTAATCC |
| 25 | AGGCAGAATAGATCGC | CCTACGGGNGGCWGCAG | GACTACHVGGGTATCTAATCC |
| 26 | AGGCAGAACTCTCTAT | CCTACGGGNGGCWGCAG | GACTACHVGGGTATCTAATCC |
| 28 | AGGCAGAAGTAAGGAG | CCTACGGGNGGCWGCAG | GACTACHVGGGTATCTAATCC |
| 29 | AGGCAGAAACTGCATA | CCTACGGGNGGCWGCAG | GACTACHVGGGTATCTAATCC |
| 31 | AGGCAGAAACACACTG | CCTACGGGNGGCWGCAG | GACTACHVGGGTATCTAATCC |
| 32 | AGGCAGAAACACGTCA | CCTACGGGNGGCWGCAG | GACTACHVGGGTATCTAATCC |

Table S2 Body weight at day 0 and day 28 of acclimation

| Body weight | CH | CS | WH | WS | Cold | Huddling | Interaction |
| --- | --- | --- | --- | --- | --- | --- | --- |
| Day 0 (g) | 47.9±2.2 | 57.9±2.8 | 40.5±2.0 | 56.5±2.5 | ns | 0.001 | ns |
| Day 28 (g) | 50.4±2.3 | 58.9±2.6 | 43.7±1.9 | 60.4±2.6 | ns | ns | ns |
| Weight gain (%) | 5.6±2.2 | 2.2±1.6 | 8.9±2.7 | 7.5±2.4 | 0.067 | ns | ns |

The body weight on day 28 of acclimation was analyzed by two-way ANCOVA with body weight on day 0 as a covariate. CH, cold huddling; CS, cold separated; WH, warm huddling; WS, warm separated; ns, not significant.

Table S3 Alpha diversity metrics table including Shannon, chao1, goods coverage and PD whole tree

| Sample ID | Shannon | chao1 | goods_coverage | PD_whole_tree |
| --- | --- | --- | --- | --- |
| 24 | 9.55397 | 11601.42 | 0.79225 | 162.5124 |
| 22 | 10.16692 | 12810.38 | 0.762833 | 184.0021 |
| 31 | 10.09865 | 12611.49 | 0.770917 | 180.6257 |
| 11 | 9.489718 | 12527.67 | 0.783 | 163.5783 |
| 21 | 9.979145 | 12483.51 | 0.767667 | 175.0014 |
| 26 | 9.644575 | 9853.444 | 0.807917 | 164.96 |
| 20 | 10.20016 | 13085.63 | 0.763583 | 179.0016 |
| 3 | 9.967471 | 12642.82 | 0.76675 | 185.2045 |
| 17 | 9.836431 | 12949.75 | 0.772583 | 175.1845 |
| 16 | 10.08572 | 12871.06 | 0.76325 | 183.7705 |
| 25 | 9.769924 | 11238.7 | 0.792917 | 166.6925 |
| 19 | 10.1512 | 14709.48 | 0.761667 | 183.235 |
| 13 | 9.869945 | 12270.04 | 0.777417 | 180.0795 |
| 32 | 10.10283 | 12182.78 | 0.777667 | 175.3122 |
| 6 | 10.15734 | 12393.97 | 0.763 | 186.8505 |
| 28 | 9.920707 | 11815.03 | 0.785417 | 179.7525 |
| 4 | 8.365411 | 11314 | 0.79525 | 168.6579 |
| 2 | 9.371207 | 9819.016 | 0.81525 | 159.3748 |
| 29 | 9.804266 | 12095.07 | 0.785583 | 166.7274 |
| 5 | 9.661597 | 11607.43 | 0.793 | 159.7556 |
| 10 | 10.198 | 12424.36 | 0.763 | 183.3743 |
| 7 | 9.63554 | 11811.24 | 0.780583 | 179.8786 |
| 9 | 10.0281 | 12617.16 | 0.767833 | 177.0001 |
| 12 | 10.10162 | 11648.65 | 0.775 | 171.7771 |

| 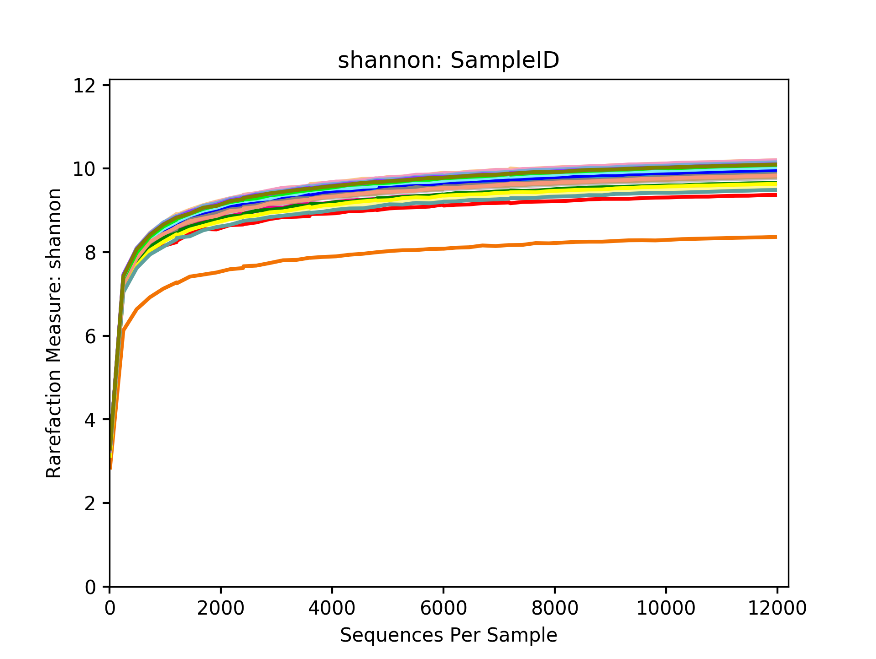  a |
| --- |
| 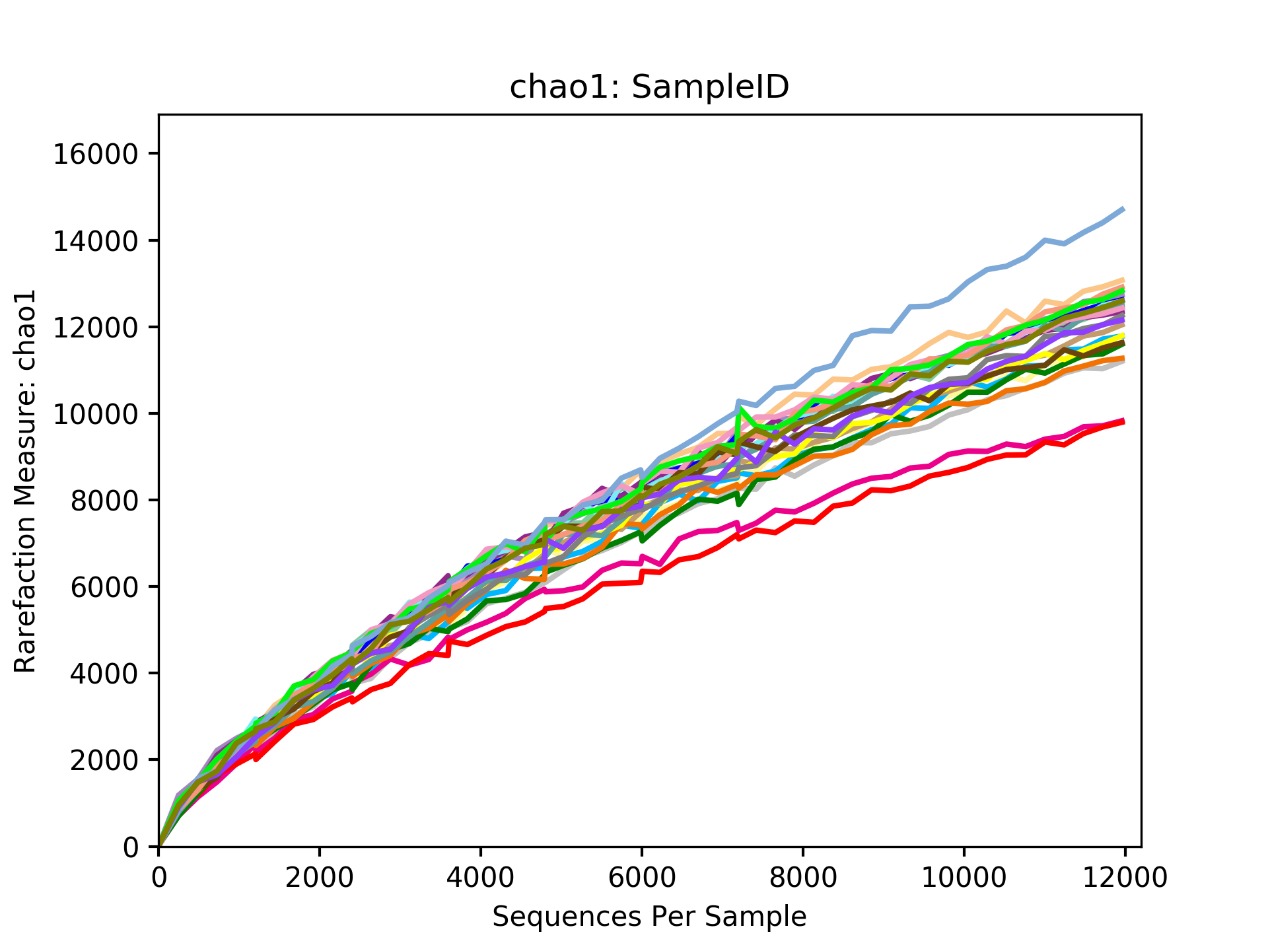  b |
| 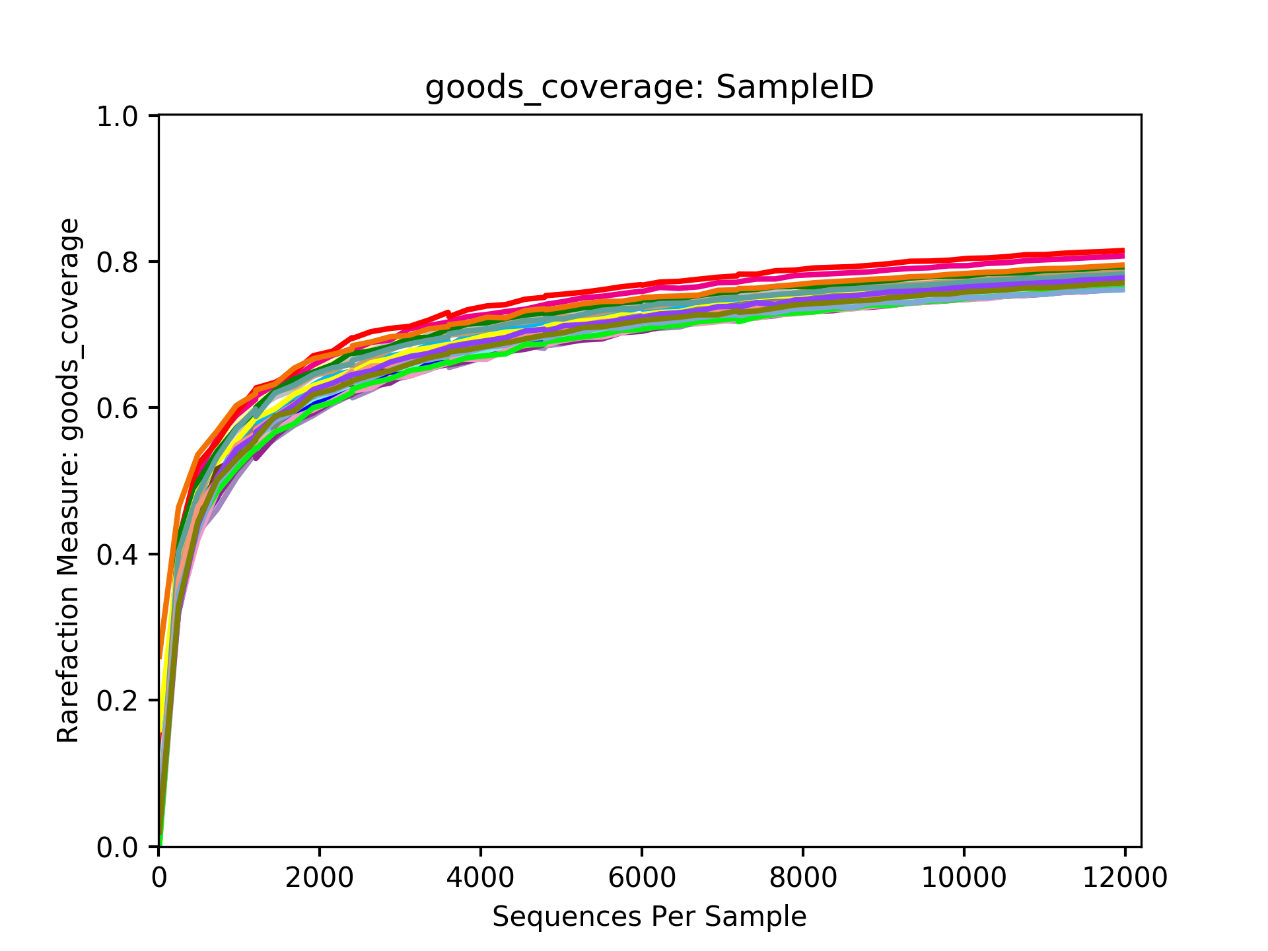  c |

Figure S1 Rarefaction curves of Shannon (A), chao1 (B) and goods coverage (C)


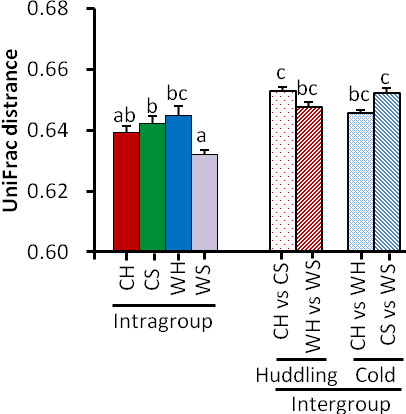


Figure S2 Mean pairwise intragroup and intergroup unweighted UniFrac distance of caecal microbiota. Bars not sharing common letters indicate a significant difference (*P* < 0.05). CH, cold huddling; CS, cold separated; WH, warm huddling; WS, warm separated.

Table S4 ADONIS results of the effect of each factor on unweighted and weighted UniFrac distance

|  | Unweighted UniFrac | | Weighted UniFrac | |
| --- | --- | --- | --- | --- |
|  | *R*^2^ | *P* | *R*^2^ | *P* |
| Cold vs Warm | 0.04901 | 0.003 | 0.06866 | 0.103 |
| Huddling vs Separated | 0.05113 | 0.001 | 0.07002 | 0.082 |
| CH vs CS vs WH vs WS | 0.15087 | 0.001 | 0.25175 | 0.003 |

CH, cold huddling; CS, cold separated; WH, warm huddling; WS, warm separated.

Table S5 Relative abundance of differential bacterial taxonomy in CH (cold huddling), CS (cold separated), WH (warm huddling) and WS (warm separated) voles (LDA > 2)

| Taxon | Type | LDA Score | CH | | CS | | WH | | WS | |
| --- | --- | --- | --- | --- | --- | --- | --- | --- | --- | --- |
|  |  |  | Mean | SEM | Mean | SEM | Mean | SEM | Mean | SEM |
| k__Bacteria|p__Firmicutes|c__Clostridia|o__Clostridiales|f__Lachnospiraceae | CH | 3.872394174 | 0.131215 | 0.010151 | 0.095997 | 0.011358 | 0.106033 | 0.008101 | 0.073926 | 0.005481 |
| k__Bacteria|p__Firmicutes|c__Clostridia|o__Clostridiales|f__Veillonellaceae | CH | 2.677850051 | 0.004731 | 0.001122 | 0.001825 | 0.000396 | 0.003396 | 0.001168 | 0.001393 | 0.000452 |
| k__Bacteria|p__TM7|c__TM7_3|o__CW040|f__F16 | CS | 2.184491536 | 0.000774 | 0.000147 | 0.001579 | 9.9E-05 | 0.001288 | 0.000125 | 0.001538 | 0.000403 |
| k__Bacteria|p__Proteobacteria|c__Gammaproteobacteria|o__Xanthomonadales|f__Sinobacteraceae | CS | 2.227167559 | 5.83E-05 | 1.89E-05 | 0.000881 | 0.000437 | 9.71E-05 | 4.32E-05 | 0.000374 | 0.000191 |
| k__Bacteria|p__Proteobacteria|c__Alphaproteobacteria|o__Rickettsiales | CS | 2.530796514 | 0.000206 | 4.68E-05 | 0.002678 | 0.001473 | 0.00105 | 0.000302 | 0.001164 | 0.000514 |
| k__Bacteria|p__Proteobacteria|c__Betaproteobacteria|o__Burkholderiales|f__Comamonadaceae | CS | 2.396918096 | 5.16E-05 | 3.22E-05 | 0.000469 | 0.000116 | 0.000157 | 7.97E-05 | 0.000333 | 0.000114 |
| k__Bacteria|p__Proteobacteria|c__Alphaproteobacteria | CS | 2.666098637 | 0.000612 | 0.000136 | 0.004053 | 0.001594 | 0.001497 | 0.000263 | 0.002711 | 0.000785 |
| k__Bacteria|p__TM7|c__TM7_3 | CS | 2.184491536 | 0.000774 | 0.000147 | 0.001579 | 9.9E-05 | 0.001288 | 0.000125 | 0.001538 | 0.000403 |
| k__Bacteria|p__Proteobacteria|c__Gammaproteobacteria|o__Xanthomonadales | CS | 2.313216646 | 9.34E-05 | 2.84E-05 | 0.001162 | 0.000567 | 0.000104 | 4.19E-05 | 0.000451 | 0.00019 |
| k__Bacteria|p__TM7|c__TM7_3|o__CW040 | CS | 2.184491536 | 0.000774 | 0.000147 | 0.001579 | 9.9E-05 | 0.001288 | 0.000125 | 0.001538 | 0.000403 |
| k__Bacteria|p__TM7 | CS | 2.184491536 | 0.000774 | 0.000147 | 0.001579 | 9.9E-05 | 0.001288 | 0.000125 | 0.001538 | 0.000403 |
| k__Bacteria|p__Firmicutes|c__Clostridia|o__Clostridiales|f__Clostridiaceae | WH | 2.323466701 | 0.00133 | 0.000137 | 0.001255 | 0.000195 | 0.002644 | 0.000777 | 0.002611 | 0.000241 |
| k__Bacteria|p__Bacteroidetes|c__Bacteroidia|o__Bacteroidales|f__Porphyromonadaceae | WH | 3.004875972 | 4.2E-05 | 1.75E-05 | 3.05E-05 | 1.06E-05 | 0.000124 | 2.98E-05 | 0.000116 | 2.87E-05 |
| k__Bacteria|p__Proteobacteria|c__Alphaproteobacteria|o__Rhizobiales | WS | 2.800712094 | 6.58E-05 | 3.63E-05 | 0.00015 | 1.75E-05 | 6.05E-06 | 6.05E-06 | 0.00019 | 6.32E-05 |
| k__Bacteria|p__Bacteroidetes|c__Bacteroidia|o__Bacteroidales | WS | 3.86103889 | 0.147831 | 0.008413 | 0.124294 | 0.025506 | 0.129205 | 0.017718 | 0.187729 | 0.007322 |
| k__Bacteria|p__Firmicutes|c__Bacilli|o__Lactobacillales|f__Lactobacillaceae|g__Lactobacillus | WS | 3.860428019 | 0.017849 | 0.001872 | 0.015399 | 0.001811 | 0.015619 | 0.001632 | 0.072648 | 0.005979 |
| k__Bacteria|p__Proteobacteria|c__Alphaproteobacteria|o__Rhizobiales|f__Hyphomicrobiaceae | WS | 2.86499842 | 1.76E-05 | 1.3E-05 | 5.13E-05 | 1.92E-05 | 0 | 0 | 9.09E-05 | 5.1E-05 |
| k__Bacteria|p__Firmicutes|c__Bacilli|o__Lactobacillales|f__Lactobacillaceae | WS | 3.860428019 | 0.017849 | 0.001872 | 0.015399 | 0.001811 | 0.015619 | 0.001632 | 0.072648 | 0.005979 |
| k__Bacteria|p__Tenericutes|c__Mollicutes | WS | 2.343051786 | 0.000385 | 9.16E-05 | 0.000661 | 0.000129 | 0.000624 | 0.000159 | 0.001056 | 0.000163 |
| k__Bacteria|p__Acidobacteria|c__Sva0725|o__Sva0725 | WS | 2.342428849 | 0 | 0 | 3.87E-05 | 1.57E-05 | 0.000157 | 0.000157 | 0.000183 | 0.000123 |
| k__Bacteria|p__Bacteroidetes | WS | 3.860960886 | 0.147858 | 0.008398 | 0.124357 | 0.025493 | 0.12924 | 0.017717 | 0.187775 | 0.007321 |
| k__Bacteria|p__Cyanobacteria|c__4C0d_2|o__YS2 | WS | 2.568223242 | 0.002118 | 0.000469 | 0.004498 | 0.000776 | 0.002643 | 0.000573 | 0.004678 | 0.000436 |
| k__Bacteria|p__Bacteroidetes|c__Bacteroidia | WS | 3.86103889 | 0.147831 | 0.008413 | 0.124294 | 0.025506 | 0.129205 | 0.017718 | 0.187729 | 0.007322 |
| k__Bacteria|p__Tenericutes|c__Mollicutes|o__RF39 | WS | 2.468631396 | 0.000262 | 7.08E-05 | 0.000414 | 0.000115 | 0.000318 | 8.82E-05 | 0.000923 | 0.000138 |
| k__Bacteria|p__Bacteroidetes|c__Bacteroidia|o__Bacteroidales|f___Odoribacteraceae_|g__Butyricimonas | WS | 3.029313081 | 7.88E-05 | 3.67E-05 | 6.69E-05 | 2.38E-05 | 9.26E-05 | 2.94E-05 | 0.000246 | 4.78E-05 |
| k__Bacteria|p__Firmicutes|c__Clostridia|o__Clostridiales|f__Lachnospiraceae|g__Butyrivibrio | WS | 2.525157068 | 0.000199 | 2.89E-05 | 0.000186 | 4.72E-05 | 0.00012 | 2.01E-05 | 0.000461 | 0.000134 |
| k__Bacteria|p__Tenericutes | WS | 2.304363322 | 0.0004 | 9.24E-05 | 0.000671 | 0.000125 | 0.000646 | 0.000165 | 0.001206 | 0.000222 |
| k__Bacteria|p__Firmicutes|c__Clostridia|o__Clostridiales|f__Clostridiaceae|g__Clostridium | WS | 2.347915285 | 0.000868 | 0.000195 | 0.000913 | 0.00022 | 0.002211 | 0.000763 | 0.002267 | 0.000224 |
| k__Bacteria|p__Acidobacteria|c__Sva0725 | WS | 2.336954245 | 0 | 0 | 3.87E-05 | 1.57E-05 | 0.000157 | 0.000157 | 0.000183 | 0.000123 |
| k__Bacteria|p__Firmicutes|c__Clostridia|o__Clostridiales|f__Peptococcaceae | WS | 2.458035768 | 0.000484 | 9.01E-05 | 0.000568 | 5.9E-05 | 0.000857 | 4.45E-05 | 0.000944 | 0.000167 |
| k__Bacteria|p__Firmicutes|c__Bacilli|o__Lactobacillales | WS | 3.860604154 | 0.01838 | 0.001911 | 0.015789 | 0.001844 | 0.016039 | 0.001688 | 0.073063 | 0.006 |
| k__Bacteria|p__Cyanobacteria|c__4C0d_2 | WS | 2.57164205 | 0.002129 | 0.000466 | 0.004536 | 0.000785 | 0.002643 | 0.000573 | 0.004678 | 0.000436 |
| k__Bacteria|p__Firmicutes|c__Bacilli | WS | 3.863303832 | 0.018752 | 0.001816 | 0.016045 | 0.001799 | 0.016065 | 0.001686 | 0.073493 | 0.006081 |
| k__Bacteria|p__Bacteroidetes|c__Bacteroidia|o__Bacteroidales|f__S24_7 | WS | 3.887843229 | 0.129548 | 0.008745 | 0.091825 | 0.019386 | 0.106491 | 0.013993 | 0.157253 | 0.009675 |

| 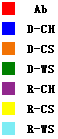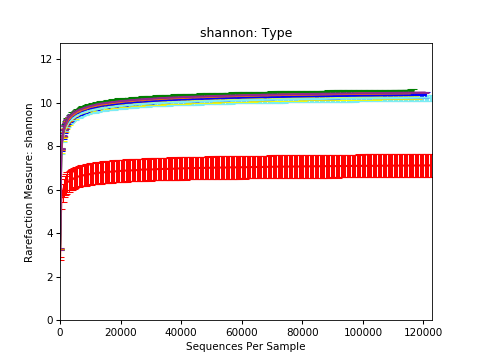  a | 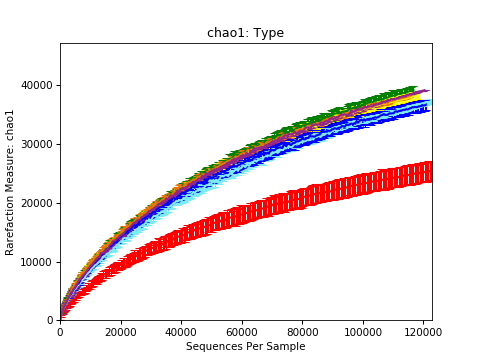  b |
| --- | --- |
| 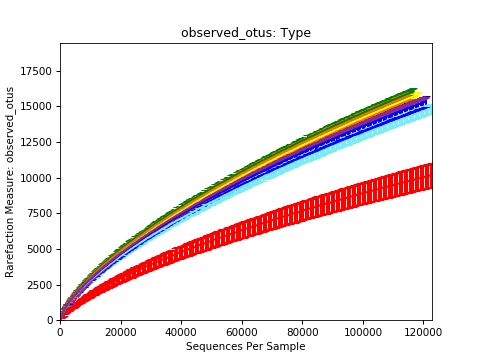  c  d | 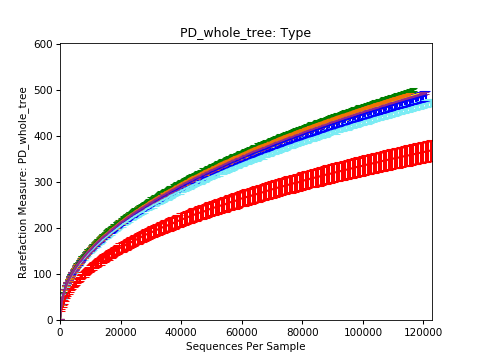 |

Figure S3 Rarefaction curves of Shannon (a), chao1 (b), observed OTUs (c) and PD whole tree (d) in CMT experiment.

Ab, antibiotics; D, donors; R, recipients; CH, cold huddling; CS, cold separated; WS, warm separated.





Figure S4 The diversity and composition of caecal microbiota in CMT experiment

(A-C) PCoA plots based on unweighted UniFrac distance. (D) Differential bacterial taxonomy selected by LEfSe analysis with LDA score > 2 in caecal microbiota community. (E-J) Relative abundance of *Christensenellaceae*, *Desulfovibrionales*, *Helicobacter*, *Paludibacter*, RF39 and *Tenericutes*. Ab, antibiotics; D, donors; R, recipients; CH, cold huddling; CS, cold separated; WS, warm separated.
